# CHARACTERIZING CAREGIVER NEGLECT IN OLDER PERSONS WITH DEMENTIA: OPERATIONAL DEFINITIONS AND RISK FACTORS

**DOI:** 10.1093/geroni/igad104.0452

**Published:** 2023-12-21

**Authors:** E-Shien Chang, Jerad Moxley, Karl Pillemer, Mark Lachs, Tony Rosen, Sara Czaja

**Affiliations:** Weill Cornell Medicine, New York City, New York, United States; Weill Cornell Medicine, New York City, New York, United States; Cornell University, Ithaca, New York, United States; Weill Cornell Medicine, New York City, New York, United States; Weill Cornell Medicine, New York City, New York, United States; Weill Cornell Medicine, New York City, New York, United States

## Abstract

Neglect by caregivers, a combination of behaviors through acts of omission or commission, is one of the most widespread forms of elder mistreatment. Examining varying definitions and thresholds of caregiver neglect may yield insights to inform intervention development. Our data were drawn from 244 dyads of racially/ethnically diverse dementia caregivers (CG) and care-recipients (CR). Informed by prior research, we developed three definitional thresholds of caregiver neglect: (1) CR’s needs were unmet by CG (least restrictive); (2) CR’s needs were unmet by CG and there was no additional informal care (moderate restrictive); and (3) CR’s needs were unmet by CG and there was no additional informal or formal care (most restrictive). Variables selected for multivariate logistic regression models examining predictors of neglect were based on significance in bivariate analysis (p< 0.10). One-week prevalence of caregiver neglect varied according to the three thresholds (43% to 11%). Sociodemographic characteristics of neglect differed across thresholds in bivariate analyses. The caregiver being male (OR=4.1, 95%CI=1.68-10.53) and being African American (OR= 2.12, 95%CI=1.01-4.54) was significantly associated with greater risk of neglect but only in the model predicting the least restrictive neglect. In models predicting moderate and most restrictive neglect, greater CG social support was associated with lower risk of neglect (OR=0.95, 95%CI=0.90-0.99; OR=0.93, 95%CI=0.89-0.98; respectively). Our findings indicate that caregiver neglect was common in our sample and that social support may play a key role in alleviating risk of neglect. Our analysis demonstrates important nuances in predictors of neglect by operationalizing neglect as a multifactorial phenomenon.

